# Deaths

**Published:** 1875-08

**Authors:** 


*Deaths.*

ELIZA BONSAEK POAGUE, infant daughter of Dr. Jno. A. and Eliza B. Poague, died February 1, 1875, aged eight weeks.

MRS. ELIZA B. POAGUE, wife of Dr. Jno. A. Poague, and daughter of Jacob Bonsaek, Esq., of Roanoke county, Va., died at her home in Rockbridge county, Va., January 4, 1875, in the 24th year of her age.

Our medical brother has our deepest sympathy in his most sad and great bereavement, though his is an affliction where words of condolence seem but an empty sound, and have no "heart's-ease" in their apparently cold and meaningless expression. May he find sufficient and abiding consolation in the Divine sympathy of that "MAN of sorrows, acquainted with grief;" and while he thinks, in tears and sadness, of the beloved young wife "under the daises" and her little babe gently sleeping by her side, would fain exclaim in the bitterness of his bereavement:

"'Alas! for love, if thou wert all,
And nought beyond, O Earth.''

May he yet feel, indeed, that "Earth has no sorrow that Heaven cannot heal." P.

